# Rapid Visual Detection of Senecavirus A Based on RPA-CRISPR/Cas12a System with Canonical or Suboptimal PAM

**DOI:** 10.3390/v17091264

**Published:** 2025-09-18

**Authors:** Xinrui Zhao, Genghong Jiang, Qinyi Ruan, Yunjie Qu, Xiaoyu Yang, Yongyan Shi, Dedong Wang, Jianwei Zhou, Jue Liu, Lei Hou

**Affiliations:** 1 Department of Preventive Veterinary Medicine, College of Veterinary Medicine, Yangzhou University, No. 48 Wenhui Road, Hanjiang District, Yangzhou 225009, China; 222001134@stu.yzu.edu.cn (X.Z.); jiang20220827@163.com (G.J.); rqy15952995997@163.com (Q.R.); yunjieequ@163.com (Y.Q.); yxiaoyu0512@163.com (X.Y.); syy2213733998@163.com (Y.S.); wddyzu@163.com (D.W.); jwzhou@yzu.edu.cn (J.Z.); liujue@yzu.edu.cn (J.L.); 2 Jiangsu Co-Innovation Center for Prevention and Control of Important Animal Infectious Diseases and Zoonoses, Yangzhou University, Yangzhou 225009, China

**Keywords:** Senecavirus A (SVA), recombinant polymerase amplification (RPA), CRISPR/Cas12a, one-pot assay, suboptimal PAM

## Abstract

Senecavirus A (SVA) is an emerging pathogen responsible for vesicular lesions and neonatal mortality in swine. In the absence of effective vaccines or therapeutics, early and accurate diagnosis is essential for controlling SVA outbreaks. Although nucleic acid-based detection methods are commonly employed, there remains a pressing need for rapid, convenient, highly sensitive, and specific diagnostic tools. Here, we developed a two-pot assay combining recombinase polymerase amplification (RPA) with CRISPR/Cas12a containing crRNA targeting canonical protospacer adjacent motifs (PAMs) for simple, rapid, and visual identification of SVA in clinical samples. Subsequently, we successfully streamlined this system into a one-pot assay by selecting a specially designed crRNA targeting suboptimal PAM and integrating RPA amplification reagents and CRISPR/Cas12a detection components into a single reaction system in one tube. The developed methods exhibited diagnostic specificity, showing no cross-reactivity with four major swine viruses, while showing remarkable sensitivity with a lower detection limit of just two copies. Clinical validation in field samples using these two methods revealed perfect agreement (100% concordance) with conventional quantitative PCR (qPCR) results (sample size, *n* = 28), with both assays completing detection within 30 min. These results demonstrate that both the one-pot and two-pot RPA-CRISPR/Cas12a assays offer a reliable and efficient method for detecting SVA in this pilot study. Despite the limited sample size, the assays combine rapid reaction time with high sensitivity and specificity, showing great potential for future diagnostic applications.

## 1. Introduction

Senecavirus A (SVA) is a single-stranded, positive-sense, non-enveloped RNA virus and the sole member of the genus *Senecavirus* within the family *Picornaviridae* [1]. It was first accidentally identified in 2002 as a contaminant in PER.C6 cells and was initially named Seneca Valley virus 001 (SVV-001) [2]. By 2007, SVA had been recognized as the causative agent of porcine idiopathic vesicular disease (PIVD) [3]. As an emerging pathogen, SVA is associated with vesicular lesions in pigs and increased mortality in neonatal piglets [4]. The virus was first detected in Guangdong Province, China, in 2015, and has since been reported in several other provinces [5,6,7]. The lack of specific antiviral therapies or commercially available vaccines complicates the control of SVA infections, contributing to its continued spread and posing a significant threat to the global swine industry.

Current diagnostic approaches for SVA infections mainly include serological assays and pathogen-based detection methods. Serological tests—such as serum neutralization tests and enzyme-linked immunosorbent assays (ELISAs) [8,9,10]—allow high-throughput screening of clinical samples. However, their effectiveness depends on the host’s antibody response, which limits utility for early-stage detection. In contrast, nucleic acid amplification tests (NAATs) provide clinically valuable capabilities for early diagnosis of SVA [11,12], despite their dependence on costly instrumentation and specialized operational expertise.

The Clustered Regularly Interspaced Short Palindromic Repeats (CRISPR) and CRISPR-associated protein (Cas) system has been widely used for detecting various pathogens, including viruses, bacteria, and parasites [13,14,15]. Guided by a CRISPR RNA (crRNA), the Cas12a–crRNA complex binds to and cleaves target DNA sequences containing a protospacer-adjacent motif (PAM) complementary to the crRNA spacer. Upon binding, Cas12a exhibits trans-cleavage activity, nonspecifically degrading nearby single-stranded DNA (ssDNA) [16,17]. This activity can be monitored using fluorophore–quencher-labeled ssDNA reporters, enabling real-time detection [18].

Among various CRISPR/Cas systems, Cas12a has become a powerful tool for building highly specific nucleic acid detection platforms. Conventional Cas12a-based detection requires the addition of pre-amplified nucleic acid products and a crRNA targeting a canonical PAM sequence [19,20], typically performed as a two-step (or “two-pot”) assay. However, the multi-step nature of this approach increases both operational complexity and the risk of contamination, limiting its practical applicability. Recently, a one-pot assay has been developed that integrates both amplification and CRISPR detection in a single reaction system by exploiting suboptimal PAM sequences (modified versions of the canonical PAM). This design slows reaction kinetics, reduces template consumption, and improves amplification efficiency, thereby enhancing overall assay performance [21].

Despite its high specificity, the CRISPR/Cas system still lacks the sensitivity required for clinical diagnostics. To overcome this limitation, it is often coupled with nucleic acid amplification methods such as recombinase polymerase amplification (RPA) [22,23]. Unlike PCR, RPA operates at a constant temperature, enabling rapid nucleic acid amplification without the need for sophisticated instrumentation, making it well suited for point-of-care applications.

So far, two main methods have been developed for SVA detection using recombinase polymerase amplification and CRISPR/Cas systems: the one-pot method and the two-pot method [24,25]. The two-pot approach, consistent with conventional procedures, involves transferring the products of recombinase polymerase amplification into a separate tube containing the CRISPR reaction mixture to initiate the detection step [25]. In contrast, the key advantage of the one-pot method lies in its integration of both recombinase polymerase amplification and CRISPR/Cas12a-mediated SVA detection within a single tube. This is achieved by pre-loading the CRISPR detection reagents into the cap of the reaction tube, while the components of recombinase polymerase amplification are placed at the bottom. After amplification, the tube is centrifuged to combine the two mixtures, thereby triggering the CRISPR/Cas12a detection reaction [24]. To further streamline the operational procedure by eliminating the centrifugation step, we propose the introduction of unrestricted crRNA targeting suboptimal PAM to develop a novel one-pot method for SVA detection. This approach involves pre-mixing both the recombinase polymerase amplification and CRISPR reaction components within a single tube.

In this study, we integrated recombinase polymerase amplification (RPA) with CRISPR/Cas12a to develop a two-pot detection method featuring a dual reaction system for rapid visual detection of SVA. Building on this foundation, we further optimized the system by designing an unrestricted crRNA targeting a suboptimal PAM site, thereby establishing a one-pot detection method utilizing a single reaction system that combines both RPA and CRISPR/Cas12a.

The entire reaction of these two methods can be completed within 30 min at 37 °C and has a detection limit of two viral copies and 100% concordance with quantitative PCR results, facilitating timely and accurate diagnosis of SVA. While the clinical validation included samples from diverse pig farms, the relatively small sample size (*n* = 28) remains a limitation. Studies involving larger and more varied sample sets are warranted to further validate the robustness and reliability of the assay under real-world clinical conditions.

## 2. Materials and Methods

### 2.1. Viruses, Antibodies, Plasmids, and Clinical Samples

Senecavirus A (SVA), porcine epidemic diarrhea virus (PEDV), and porcine deltacoronavirus (PDCoV) were preserved in our laboratory. Porcine reproductive and respiratory syndrome virus (PRRSV) and classical swine fever virus (CSFV) viral RNA genomes were kindly supplied by Nanhua Chen and Chengcheng Zhang (Yangzhou University), respectively. The following antibodies were obtained commercially: mouse anti-His antibodies (ab18184; Abcam, Cambridge, MA, USA) and horseradish peroxidase (HRP)-conjugated anti-mouse secondary antibodies (A9044; Sigma-Aldrich, St. Louis, MO, USA). The pCMV-HA-SVA *VP1* plasmid was reserved in our laboratory. The clinical tissue samples (spleen, lung, kidney, tonsil, colorectal, heart, liver, bronchial lymph node, submandibular lymph node, and inguinal lymph node) were collected from multiple commercial pig farms.

### 2.2. The Expression and Purification of Cas12a Protein

The Cas12a protein was expressed in *E. coli* BL21(DE3) cells transformed with the pET-28a-Cas12a plasmid, then purified using a His-tag protein purification kit (DP101-01; Transgen Biotech Co, Beijing, China), as previously described [19].

### 2.3. Viral cDNA Preparation of Various Viruses and Clinical Samples

Total RNA was extracted from supernatants containing SVA, PEDV, and PDCoV using TRIzol reagent (15596018; Invitrogen, Carlsbad, CA, USA) following the manufacturer’s protocol. Subsequently, cDNA was synthesized from the extracted total RNA using the Vazyme cDNA Synthesis Kit (R323-01; Vazyme, Nanjing, China).

### 2.4. Design and Synthesization of crRNAs and ssDNA

The crRNAs targeting the SVA *VP1* gene (GenBank Accession: MG983756.1) were designed using the online tool CHOPCHOP (https://chopchop.cbu.uib.no/) based on the Cas12a-recognized canonical PAM (TTTN) or suboptimal PAM (TNTN) and synthesized by GENEWIZ (Table S1). Additionally, the following components were commercially synthesized by GENEWIZ (Table S1): the known-target DNA and crRNA (KT-DNA and KT-crRNA) [20] and ssDNA probe linked to the fluorescent 6-FAM group and the quenching BHQ-1 group (ssDNA-FQ probe).

### 2.5. Evaluation of Cas12a Endonuclease Activity

The reaction system contained the following major components: 1 µL purified Cas12a protein (0.5 μg/μL), 1 µL target DNA (KT-DNA; 2 × 10^9^ copies/µL), 1 µL specific crRNA (KT-crRNA; 1 µM), 1 µL ssDNA-FQ reporter probe (10 µM), and 1× NEB assay buffer (B6002V; New England Biolabs, Ipswich, Massachusetts, USA). The mixture was incubated at 37 °C for 30 min to assess Cas12a endonuclease activity based on the fluorescence signals.

### 2.6. The Optimization of crRNA and Cas12a Concentrations

The Cas12a and crRNA concentrations in the CRISPR/Cas12a-SVA assay were optimized based on the following reaction system, including 1 µL purified Cas12a protein (0.5 μg/μL) with various concentrations (0.25, 0.5, or 1 μg/μL), 1 µL crRNA with various concentrations (0.25, 0.5, or 1 μM), 1 μL SVA *VP1* DNA (2 × 10^9^ copies/μL), and 1 μL ssDNA-FQ probe (10 μM) in assay buffer. The mixture was incubated at 37 °C for 30 min to determine the optimal concentrations of Cas12a and crRNA based on the fluorescence signals.

### 2.7. The Optimization of Recombinant Polymerase Amplification (RPA)

We designed three primer pairs (Table S1) targeting conserved regions of the SVA *VP1* gene (GenBank: MG983756.1) for recombinase polymerase amplification (RPA) and amplified it using the TwistAmp Basic kit (TABAS03KIT, TwistDX, Maidenhead, UK) at 37 °C for 20 min. The total volume of the reaction system is 50 μL, including 30 μL of buffer, 2.5 μL each of forward and reverse primer (10 μM), 1 μL of SVA *VP1* DNA template (2 × 10^9^ copies/μL), 2.5 μL of Magnesium Acetate (MgOAC, 280 nM), and the appropriate sterile water.

### 2.8. The Optimization of Sensitivity and Time in CRISPR/Cas12a-SVA Detection

The 50 µL reaction mixture in the CRISPR/Cas12a reaction system was assembled with the following components: Cas12a protein (0.5 ug/µL, 1 µL), crRNA (0.5 µM), ssDNA-FQ probe (10 µM), NEB Buffer (5 µL), SVA *VP1* DNA, and appropriate sterile water. The reaction was performed at 37 °C for 30 min in the presence of SVA *VP1* DNA (concentrations from 2 × 10^9^ to 2 × 10^6^ copies/µL, 1 µL) to analyze sensitivity or carried out at 37 °C for 5, 10, 20, 30, and 40 min in the presence of SVA *VP1* DNA (2 × 10^9^ copies/µL, 1 µL) to optimize reaction time based on the fluorescence signals. The copy numbers of pCMV-HA-SVA *VP1* plasmid were calculated as follows: DNA copy numbers = (6.02 × 10^23^) × (DNA concentration (ng/µL) × 10^−9^)/(DNA length × 660 Da/bp).

### 2.9. Sensitivity and Specificity of Two-Pot RPA-CRISPR/Cas12a-SVA Detection Method

The RPA reaction containing 1 μL 10-fold serially diluted SVA *VP1* DNA (concentrations 2 × 10^11^, 2 × 10^9^, 2 × 10^7^, 2 × 10^5^, 2 × 10^3^, 2 × 10^1^, and 2 × 10^0^ copies/µL, respectively) or 1 μL cDNA of PDCoV, PRRSV, SVA, CSFV, and PEDV was performed at 37 °C for 20 min. Following amplification, 5 µL of RPA products were added to the CRISPR/Cas12a detection system, and the sensitivity and specificity were subsequently evaluated through fluorescence signal analysis.

### 2.10. Establishment and Sensitivity of One-Pot RPA-CRISPR/Cas12a-SVA Detection Method

The 30 µL reaction mixture in the one-pot CRISPR/Cas12a-SVA reaction system was assembled with the following optimized components: buffer (15 µL), forward or reverse primer (10 µM, 1.2 µL), SVA *VP1* DNA (1 µL), MgOAC (280 nM, 2 µL), ssDNA-FQ probe (1 µL), NEBuffer (3 µL), crRNA (0.5 µM, 1 µL), Cas12a (0.5 µg/µL, 1.2 µL), and appropriate sterile water. The reaction was performed at 37 °C for 30 min.

### 2.11. The Detection of Clinical Samples

A total of 28 clinical samples (200 mg/sample) were homogenized, followed by the extraction and/or reverse transcription of viral RNA, and then detected by qPCR using the reported primers [26] and one- or two-pot RPA-CRISPR/Cas12a-SVA. All positive samples in the above detection were further confirmed by sequencing.

### 2.12. Ethics Statement

This study was approved by the Animal Ethical and Welfare Committee for Institutional Animal Care and Use Committee (IACUC) of Yangzhou University (approval code. YZU202302065; approval date. 17 February 2023).

### 2.13. Statistical Analysis

Statistical significance was determined using either one-way ANOVA or Student’s *t*-test in Prism 9.0 (GraphPad Software, Boston, MA, USA), with *p* < 0.05 considered statistically significant.

## 3. Results

### 3.1. Endonuclease Activity Assay of Purified Cas12a Protein

**Figure 1:**
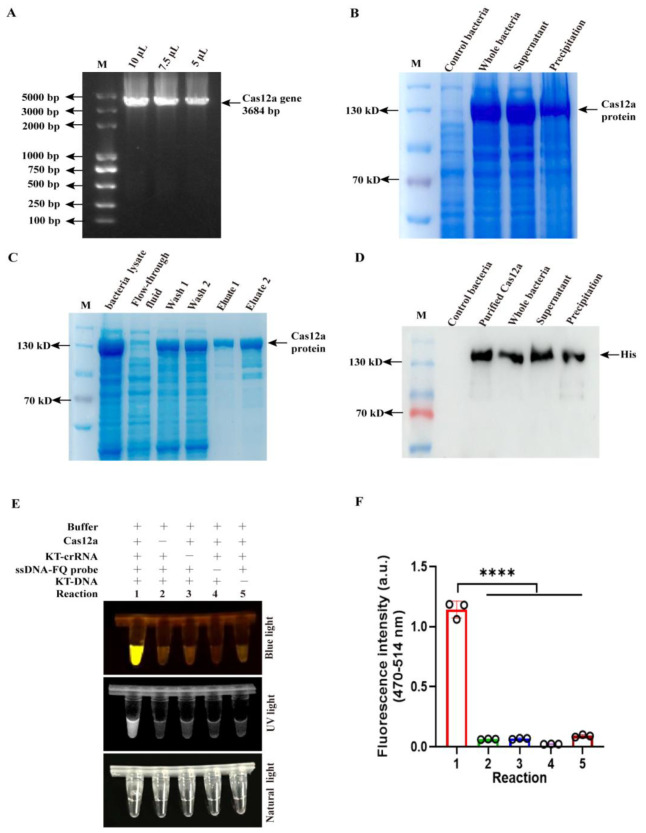
Endonuclease activity evaluation of purified Cas12a. (**A**) The Cas12a gene was amplified from a 6×His-MBP-TEV-huLbCas12a-encoding plasmid. (**B**) The whole bacteria, supernatant, and precipitation of recombinant bacteria were fractioned by sodium dodecyl sulfate-polyacrylamide gel electrophoresis (SDS-PAGE) and stained by Coomassie blue. (**C**) Cas12a proteins were eluted using buffers containing imidazole at concentrations of 50 mM (Wash 1), 100 mM (Wash 2), 200 mM (Eluate 1), and 300 mM (Eluate 2), respectively. (**D**) The whole bacteria, supernatant, and precipitation of recombinant bacteria and purified proteins (Eluate proteins) were analyzed by Western blotting with anti-His antibody. (**E**,**F**) The assay of purified Cas12a endonuclease activity via blue light, UV light (**E**), and fluorescence signal (**F**) in CRISPR/Cas12a reaction (*n* = 3 technical replicates). The data are expressed as the means ± SDs from three independent experiments (**** *p* < 0.0001).

The Cas12a gene was amplified from a 6×His-MBP-TEV-huLbCas12a-encoding plasmid (Figure 1A) and subsequently cloned into the pET28a expression vector. Using a prokaryotic expression system, we successfully expressed and purified Cas12a proteins from the soluble fraction of bacterial lysates, as confirmed by SDS-PAGE and Western blot analysis (Figure 1B–D).

To assess the endonuclease activity of the purified Cas12a proteins, we conducted an in vitro cleavage assay under RNase-free conditions. The reaction mixture contained purified Cas12a proteins, known-target DNA (KT-DNA), target-specific KT-crRNA, and a single-stranded fluorescent-quencher (ssDNA-FQ) reporter probe, incubated at 37 °C for 30 min. Fluorescence signal detection and visualization under blue/UV light demonstrated robust endonuclease activity of the purified proteins (Figure 1E,F).

### 3.2. The Optimization of Cas12a and crRNA Concentration and RPA Primers in CRISPR/Cas12a-

#### SVA Detection Method

**Figure 2:**
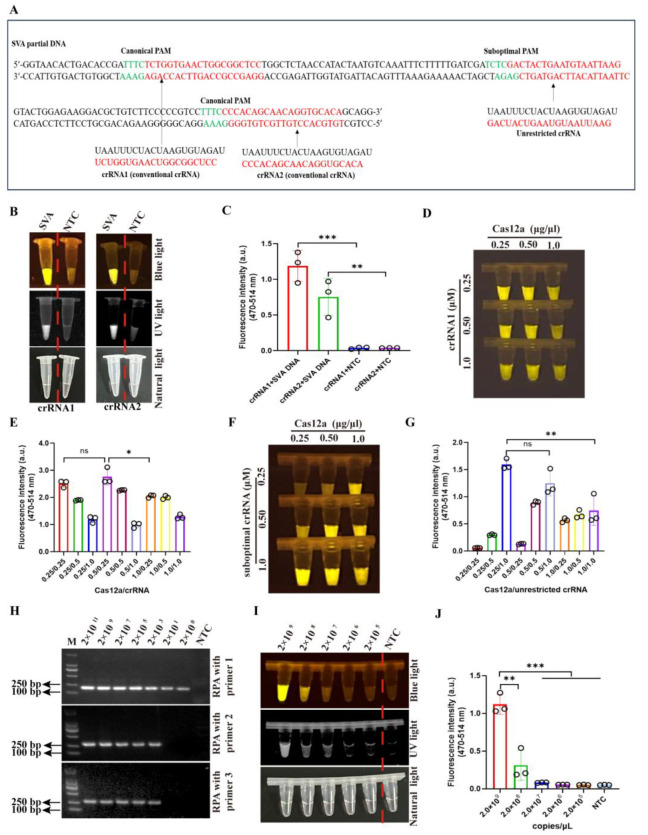
The optimization of Cas12a and crRNA concentration and RPA primer pairs in CRISPR/Cas12a reaction. (**A**) Schematic diagram of crRNAs targeting canonical and suboptimal PAM in SVA *VP1* gene partial sequence. (**B**,**C**) The assay of conventional crRNAs (crRNA1 and crRNA2) targeting canonical PAM via blue light, UV light (**B**), and fluorescence signal (**C**) in CRISPR/Cas12a reaction (*n* = 3 technical replicates). (**D**,**E**) The optimization of the concentration of crRNA targeting canonical PAM (crRNA1 and crRNA2) and Cas12a protein (**D**), and fluorescence signal (**E**) via CRISPR/Cas12a assay (*n* = 3 technical replicates). (**F**,**G**) The optimization of the concentration of the unrestricted crRNA targeting suboptimal PAM and Cas12a protein (**F**) and fluorescence signal (**G**) via CRISPR/Cas12a assay (*n* = 3 technical replicates). (**H**) Screen of RPA primer pairs based on amplification efficiency for different concentrations of viral DNA. (**I**,**J**) The sensitivity assay of the CRISPR/Cas12a reaction for different concentrations of SVA DNA detection via blue light, UV light (**I**), and fluorescence signal (**J**) via CRISPR/Cas12a assay (*n* = 3 technical replicates). The data are expressed as the means ± SDs from three independent experiments (ns, *p* > 0.05; *, *p* < 0.05; **, *p* < 0.01; ***, *p* < 0.001).

Three crRNAs targeting the SVA *VP1* gene were designed and synthesized by GENEWIZ. Among these, crRNA1 and crRNA2, two conventional crRNAs, were constrained by canonical PAM (TTTN), whereas an unrestricted crRNA was designed to accommodate a suboptimal PAM (TNTN) (Figure 2A). Subsequent functional evaluation revealed that crRNA1 and crRNA2 exhibited good detection effects, with crRNA1 having a stronger fluorescence signal than crRNA2 (Figure 2B,C). Thus, the crRNA1 was selected for subsequent study. Previous reports showed that the crRNAs targeting suboptimal and canonical PAM are suitable for the one- and two-pot CRISPR/Cas12a detection method, respectively [19]. Therefore, we exploited crRNA1 to perform detection in a two-pot assay, while the unrestricted crRNA was used in a one-pot system. Through systematic optimization, the optimal reaction conditions were determined to be 0.5 µg/µL Cas12a protein and 0.25 µM crRNA1 in a two-pot assay (Figure 2D,E) and 0.25 µg/µL Cas12a protein and 1 µM unrestricted crRNA in a one-pot assay (Figure 2F,G), as validated by fluorescence-based activity assays.

The RPA-CRISPR/Cas12a-SVA detection system comprises two critical stages: RPA amplification and CRISPR/Cas12a detection. For the amplification stage, we evaluated the amplification sensitivity of three RPA primer pairs (RPA1-RPA3) targeting the conserved *VP1* sequence of SVA using ten-fold serial dilutions of SVA *VP1* DNA (concentrations from 2 × 10^11^ to 2 × 10^0^ copies/μL) and demonstrated that the RPA1 primer pair exhibited significantly higher amplification efficiency compared to RPA2 and RPA3 primer pairs (Figure 2H). For the CRISPR/Cas12a-SVA detection stage, fluorescence-based analysis revealed a detection limit of 2 × 10^8^–2 × 10^9^ copies/μL (Figure 2I,J), indicating that individual CRISPR/Cas12a detection has extremely low sensitivity.

### 3.3. Development of the Two-Pot RPA-CRISPR/Cas12a Assay for SVA Detection

**Figure 3:**
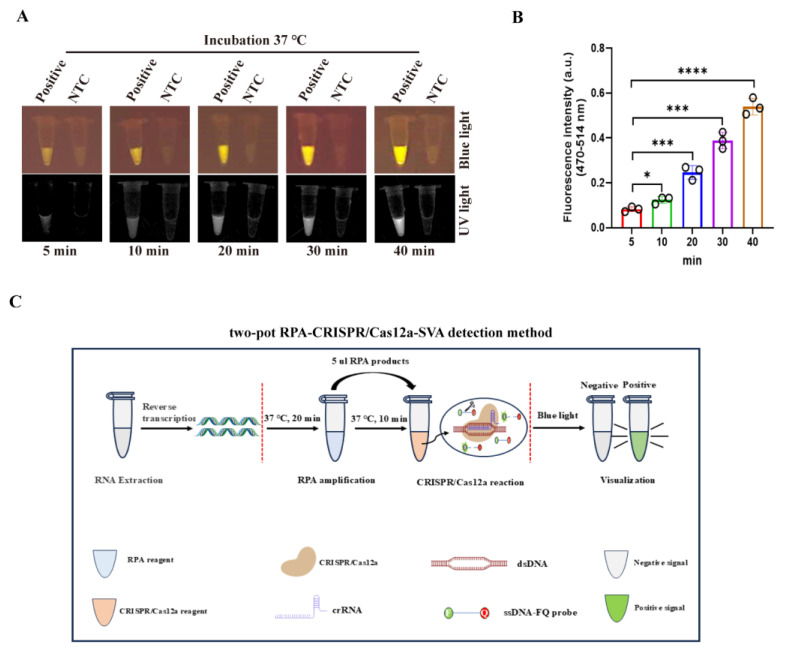
Establishment of two-pot RPA-CRISPR/Cas12a method for SVA detection. (**A**,**B**) Time-course analysis of the RPA-CRISPR/Cas12a-SVA DNA detection by blue light, UV light (**A**), and fluorescence signal (**B**) via CRISPR/Cas12a assay (*n* = 3 technical replicates). (**C**) Schematic diagram of two-pot RPA-CRISPR/Cas12a method for SVA detection. Viral RNA extracted from the sample was first reverse transcribed into cDNA. The resulting cDNA was then amplified using the RPA system at 37 °C for 20 min. Subsequently, 5 μL of the RPA product was added to the CRISPR/Cas12a reaction system and incubated for 30 min. Finally, the outcome of the reaction was detected under both blue, UV light, and fluorescence signal. The data are expressed as the means ± SDs from three independent experiments (*, *p* < 0.05; ***, *p* < 0.001; ****, *p* < 0.0001).

The two-pot RPA-CRISPR/Cas12a detection system was developed through integration of isothermal RPA amplification with CRISPR-mediated nucleic acid sensing, followed by optimization of reaction time. The RPA amplification step was performed at 20 min according to the manufacturer’s protocol and the CRISPR/Cas12a-SVA detection step was evaluated over varying durations (5, 10, 20, 30, and 40 min) using SVA *VP1* DNA (2 × 10^9^ copies/µL) as the target. Our results demonstrated that UV light, blue light, and fluorescence signals progressively enhanced during CRISPR/Cas12a-SVA detection, peaking at 40 min (Figure 3A,B). No signal was observed in the negative control. Notably, a clear fluorescence signal was detectable as early as 5 min, prompting the selection of a 10 min CRISPR reaction time to balance speed and sensitivity. Thus, the total assay time was established at 30 min, requiring 20 min for RPA amplification and as little as 10 min for CRISPR/Cas12a-mediated detection (Figure 3C).

### 3.4. Assessment of Detection Sensitivity and Target Specificity in a Two-Pot RPA-CRISPR/Cas12a-

#### SVA System

**Figure 4:**
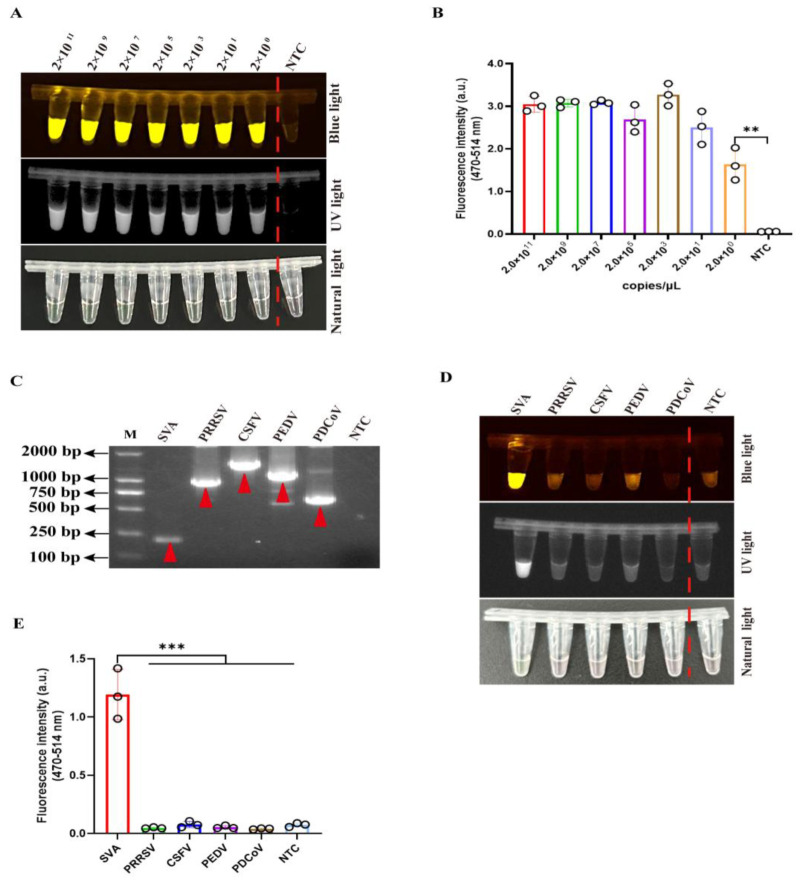
The sensitivity and specificity of two-pot RPA-CRISPR/Cas12a method for SVA detection. (**A**,**B**) Sensitivity evaluation of the two-pot RPA-CRISPR/Cas12a-SVA with serially diluted viral DNA (10-fold) by blue light, UV light (**A**), and fluorescence signal (**B**) via CRISPR/Cas12a assay (*n* = 3 technical replicates). (C) The specific PCR products (red triangle) targeting PRRSV, PEDV, PDCoV, SVA, and CSFV. (**D**,**E**) The specificity of two-pot RPA-CRISPR/Cas12a method for PRRSV, PEDV, PDCoV, SVA, and CSFV detection by blue light, UV light (**D**), and fluorescence signal (**E**) via the CRISPR/Cas12a assay (*n* = 3 technical replicates). The data are expressed as the means ± SDs from three independent experiments (**, *p* < 0.01; ***, *p* < 0.001).

The sensitivity of this two-pot detection system was evaluated using ten-fold serial dilutions of SVA *VP1* DNA (2 × 10^11^ to 2 × 10^0^ copies/μL). The detection effect was assessed through simultaneous monitoring of three signal: UV light, blue light, and fluorescence emission. The assay demonstrated remarkable sensitivity, achieving a detection limit of 2 × 10^0^ copies/μL of viral DNA (Figure 4A,B).

To further validate the specificity of this two-pot detection method, we performed comprehensive cross-reactivity testing with four common swine viruses, including porcine reproductive and respiratory syndrome virus (PRRSV), classical swine fever virus (CSFV), porcine epidemic diarrhea virus (PEDV), and porcine deltacoronavirus (PDCoV). Initial confirmation of viral RNA was achieved through conventional reverse transcription (RT)-PCR (Figure 4C and Table S1). Subsequently, the specificity evaluation demonstrated that SVA-positive samples generated specific UV light, blue light, and fluorescence signals, while all non-target viruses showed no detectable signal (Figure 4D,E). These results conclusively demonstrated that this two-pot RPA-CRISPR/Cas12a-SVA system maintains high sensitivity and specificity for SVA detection without cross-reactivity to other prevalent swine pathogens.

### 3.5. Comparative Evaluation Between RPA-CRISPR/Cas12a-Based and qPCR Methods for SVA

**Figure 5:**
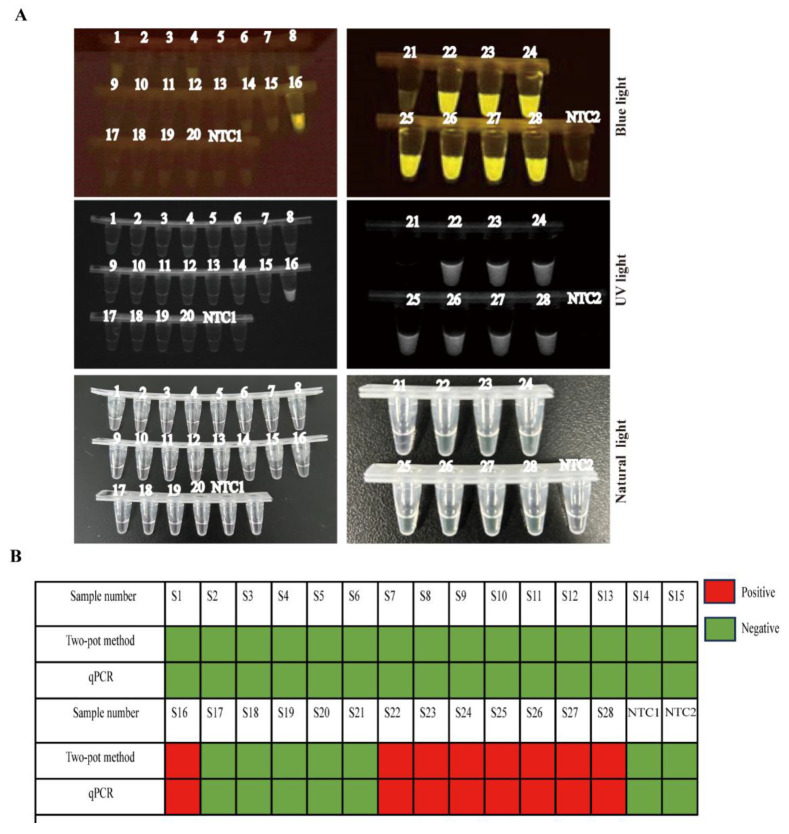
Detection of SVA field samples in two-pot RPA-CRISPR/Cas12a reaction. (**A**) Detection of SVA in 28 field samples using the two-pot RPA-CRISPR/Cas12a method with blue light and UV light. (**B**) Concordance assessment of two-pot RPA-CRISPR/Cas12a method with qPCR for SVA detection (negative = green, positive = red, and NTC = no-viral DNA control).

To evaluate clinical performance, we analyzed 28 field samples representing ten tissue types (spleen, lung, kidney, tonsil, colorectal, heart, liver, bronchial lymph node, submandibular lymph node, and inguinal lymph node). This two-pot method identified 8 positive and 20 negative samples (Table 1, Figure 5A). For detection validation, we performed parallel testing using a previously published qPCR method for SVA. As shown in Figure 5B, we observed perfect concordance (100% agreement) between both methods across all clinical samples. The consistency of this detection indicated the reliability of this two-pot RPA-CRISPR/Cas12a-SVA system for clinical SVA detection.

### 3.6. Clinical Evaluation of a One-Pot RPA-CRISPR/Cas12a Assay for SVA Detection

**Figure 6:**
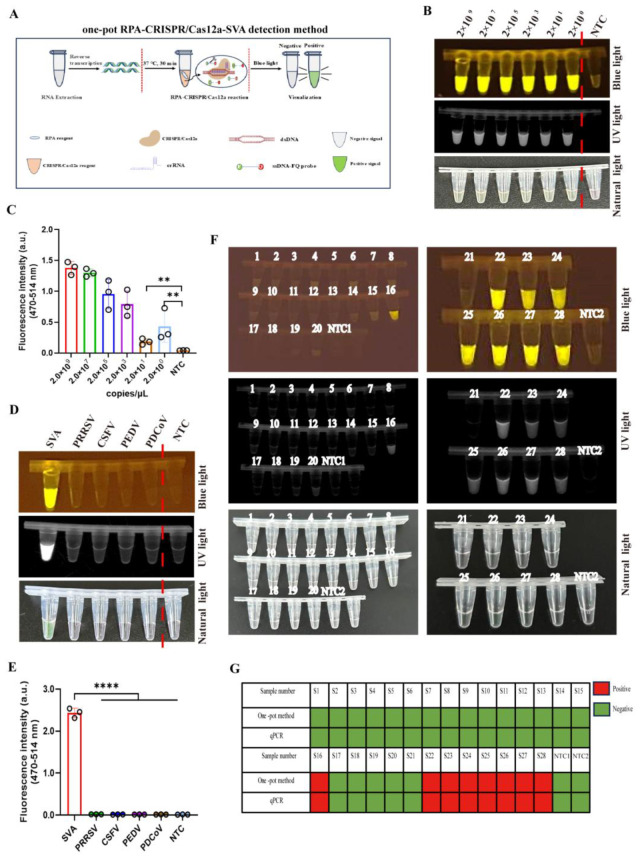
Validation of one-pot RPA-CRISPR/Cas12a method for SVA detection. (**A**) Schematic diagram of one-pot RPA-CRISPR/Cas12a method for SVA detection. The RNA extracted from the sample is first reverse transcribed into cDNA. The cDNA is then amplified and detected in a single-tube reaction system combining recombinase polymerase amplification (RPA) and the CRISPR/Cas12a system, performed at 37 °C for 30 min. Results are visualized under blue light, UV light, and fluorescence signals. (**B**,**C**) Sensitivity evaluation of the one-pot RPA-CRISPR/Cas12a-SVA with serially diluted viral DNA (10-fold) by blue light, UV light (**B**), and fluorescence signal (**C**) via CRISPR/Cas12a assay (*n* = 3 technical replicates). (**D**,**E**) The specificity of the one-pot RPA-CRISPR/Cas12a method for PRRSV, PEDV, PDCoV, SVA, and CSFV detection by blue light, UV light (**D**), and fluorescence signal (**E**) via CRISPR/Cas12a assay (*n* = 3 technical replicates). (**F**) Detection of SVA in 28 field samples using the one-pot RPA-CRISPR/Cas12a method with blue light and UV light. (**G**) Concordance assessment of one-pot RPA-CRISPR/Cas12a method with qPCR for SVA detection (negative = green, positive = red, and NTC = no-viral DNA control). The data are expressed as the means ± SDs from three independent experiments (**, *p* < 0.01; **** *p* < 0.0001).

To simplify the operation steps and reduce contamination risks, we exploited unrestricted crRNA targeting a suboptimal PAM for method development. This approach enabled successful integration of RPA amplification with CRISPR/Cas12a-SVA detection in a single-tube reaction system at the bottom of tube (Figure 6A). Remarkably, this integrated system achieved high sensitivity with a detection limit of just 2 × 10^0^ copies/μL (Figure 6B,C) and did not exhibit cross-reactivity with four common swine viruses (Figure 6D,E). Clinical detection for 28 field samples showed perfect concordance with previous results, identifying 8 positive and 20 negative cases (Figure 6F,G). These results confirmed the clinical reliability and superior performance of the one-pot detection system.

## 4. Discussion

SVA infections have occurred worldwide, severely affecting the economic viability of swine production. So far, there are no commercially available vaccine and specific drugs to protect pigs from infection. Therefore, it is necessary to develop some methods to detect SVA infection faster and more conveniently, in which the detection of pathogenic nucleic acids is the most common, such as with PCR-related techniques [11,12]. In this study, we integrated RPA amplification targeting the conserved sequence of viral *VP1* gene with CRISPR/Cas12a detection and developed one- and two-pot RPA-CRISPR/Cas12a-SVA detection methods, requiring neither costly equipment nor complicated steps.

Compared to conventional PCR-based methods, the RPA technique has experienced rapid development due to its advantages of shorter reaction time, higher sensitivity, and lower equipment requirements [27,28]. However, the possibility for non-specific amplification in RPA systems has limited its broader application [29]. To overcome this weakness, the CRISPR/Cas system is usually integrated with RPA amplification for enhancing the specificity of the RPA method [30]. In the CRISPR/Cas system, the Cas12a endonuclease, mainly targeting ssDNA for cleavage through recognition of PAM guided by crRNA, is applied more widely based on the convenience in DNA detection [31]. For example, the RPA-CRISPR/Cas 12a detection system has been successfully used to detect porcine circovirus type 3 (PCV3), African swine fever virus (ASFV), and severe acute respiratory syndrome coronavirus 2 (SARS-CoV-2) [19,32,33].

Given the mutational propensity of SVA as an RNA virus, we conducted a comprehensive analysis of SVA *VP1* gene sequences (approximately 50 SVA strains) available in the NCBI database to identify conserved regions for crRNAs design. Through systematic screening, we optimized crRNAs for efficient targeting in the CRISPR/Cas12a reaction (Figure 2). Subsequent optimization of key parameters, including Cas12a/crRNA concentration, RPA primer pairs, reaction time, specificity, and sensitivity (Figure 2, Figure 3 and Figure 4), yielded a highly efficient detection system. The developed RPA-CRISPR/Cas12a method demonstrated remarkable performance, achieving detection sensitivity as low as two copies/uL and completing the entire process within 30 min without reverse transcription step.

Building upon the conventional two-pot detection system, we successfully established an innovative single-tube reaction system (using the same RPA amplification primers as the two-pot method) that simultaneously accommodates both RPA amplification and CRISPR/Cas12a detection components in a single-tube reaction system. The key innovation of one-pot RPA-CRISPR/Cas12a detection method lies in the implementation of unrestricted crRNA targeting a suboptimal PAM, prompting the establishment of a single-tube reaction system, which is different from the published one-pot and two-pot assay [24,25], improving the simplicity of operation. Additionally, the detection limit of the one-pot detection method (two copies of the SVA genome per reaction) in our study is significantly lower compared to the published one-pot assay (10 copies of the SVA genome per reaction) [24]. Another distinct advantage of our single-tube method over previously published one-pot systems is the elimination of centrifugation steps during operation, significantly simplifying the workflow while maintaining detection efficiency. Taken together, our established one-pot method demonstrates technological innovation. A comparison between various CRISPR/Cas12a-SVA detection methods is listed in Table S2.

To validate its diagnostic utility, we applied this method to 28 field samples and observed complete concordance with RT-qPCR results (Figure 5). Collectively, we have successfully established a rapid, sensitive, and specific two-pot RPA-CRISPR/Cas12a assay for SVA detection, offering significant advantages for diagnostics. However, some limitations of this study need to be improved and optimized in the future. First, the clinical validation was based on a relatively small sample size (*n* = 28), although the sample was derived from diverse pig farms; future studies with larger and more diverse sample sets are needed to further confirm the robustness and reliability of the assay under clinical conditions. Second, given the high mutability of RNA viruses, there is a potential risk of cross-reactivity or reduced detection efficiency against emerging SVA variants. Continuous monitoring and periodic updates of the crRNA design will be essential to maintain detection accuracy. Furthermore, while the method is proposed as suitable for field use, its practical applicability—such as stability under various environmental conditions and ease of operation by non-specialists—requires more extensive validation. Lastly, while the assay is presumed to be cost-effective due to the use of low-cost instruments and straightforward technical requirements, this assertion is not yet supported by quantitative economic evidence. Future cost-benefit evaluations would strengthen its feasibility for large-scale implementation.

RPA amplification and CRISPR/Cas12a detection are two separate steps in the two-pot reaction, and this two-step process requires opening of the amplification reaction tube and transferring of the amplification product, increasing the risk of secondary operations or aerosol contamination [34]. Thus, the establishment of a one-pot detection method is urgently needed. For example, we developed a one-pot RPA-CRISPR/Cas12a-PCV3 assay with compartmentalized reagents, where tube centrifuge post-amplification initiated CRISPR detection [19]. To simplify the program and improve the coherence of detection, we further improved a one-pot assay integrating amplification and detection in a single system, using a crRNA specifically designed to target suboptimal PAM (Figure 6).

Our established one-pot method employs a single-tube reaction system—using the same RPA amplification primers as the two-pot method—to simultaneously accommodate both RPA amplification and CRISPR/Cas12a detection. A key innovation of this approach is the implementation of an unrestricted crRNA targeting a suboptimal PAM sequence, which enables a truly integrated single-tube reaction. This design differs from previously reported one-pot and two-pot assays [24,25] and significantly improves operational simplicity. Furthermore, the one-pot method developed here achieves a detection limit of two copies of SVA DNA, which is notably lower than that of a published one-pot assay (10 copies of SVA DNA) [24]. Another distinct advantage of our system is the elimination of centrifugation steps during operation, which streamlines the workflow without compromising detection efficiency. Together, these features demonstrate the technological innovation and practical advancement of our one-pot detection method.

This one-pot method achieves a detection limit of two copies of viral DNA, which is consistent with the two-pot assay. Compared to the canonical PAM, the reduced binding affinity of Cas12a for suboptimal PAM sequences attenuates its cleavage activity, thereby enhancing amplification efficiency by rebalancing the reaction dynamics [21,35]. The unrestricted crRNA we designed has certain limitations, attributed to its design within the existing RPA amplification region. If the unrestricted crRNAs are designed throughout the entire *VP1* gene sequence, the established one-pot detection method may further improve flexibility, speed, and sensitivity.

## 5. Conclusions

In conclusion, this study presents the development and validation of two detection methods—a two-pot and a one-pot assay—based on the integration of RPA and CRISPR/Cas12a for rapid, sensitive, and specific identification of SVA. The two-pot assay utilizes crRNA targeting canonical PAM sequences to enable visual detection, while the one-pot assay incorporates an unrestricted crRNA targeting a suboptimal PAM, allowing full integration of amplification and detection within a single tube. Both methods demonstrated exceptional analytical performance, achieving a detection limit as low as two copies and exhibiting no cross-reactivity with other major swine viruses. Importantly, clinical evaluations confirmed 100% concordance with standard qPCR when applied to a limited number (*n* = 28) of field samples, while reducing the total detection time to within 30 min. These results underscore the practical utility and robustness of the RPA-CRISPR/Cas12a-SVA assays, highlighting their potential as point-of-need diagnostic tools for early SVA surveillance and outbreak control in the future.

It is worth emphasizing that this technology platform exhibits excellent adaptability and extensibility. By simply replacing the crRNA and RPA primer sequence, it can be readily adapted for the detection of other swine viruses. Moreover, the detection system can be developed into a multiplex detection platform, enabling simultaneous identification and differentiation of multiple pathogens, thereby further enhancing its value in veterinary clinical practice and disease control.

## Figures and Tables

**Table 1:** Comparison between RPA-CRISPR/Cas12a and qPCR detections for clinical samples.

Sample Types	Sample Numbers	RPA-CRISPR/Cas12a	qPCR
Spleen	3	1/3	1/3
Lung	4	0/4	0/4
Kidney	2	1/2	1/2
colorectal	4	0/4	0/4
tonsil	3	1/3	1/3
heart	2	0/2	0/2
liver	4	1/4	1/4
bronchial lymph node	3	2/3	2/3
Submandibular lymph node	1	1/1	1/1
Inguinal lymph node	2	1/2	1/2
Positive rates		28.5%	28.5%

## Data Availability

The data presented in this study are available upon request from the corresponding author.

## Supplementary Materials

The following supporting information can be downloaded at: https://www.mdpi.com/article/10.3390/v17091264/s1, Table S1: Primers, crRNA, and probe in this study; Table S2: Comparison of various CRISPR/Cas12a-SVA detection methods.
