# Supplementary material for: Rapid Visual Detection of Senecavirus A Based on RPA-CRISPR/Cas12a System with Canonical or Suboptimal PAM
Source: Viruses. 2025 Sep 18;17(9):1264. doi: 10.3390/v17091264 (PMC12474415; doi:10.3390/v17091264)
Supplement: Supplementary file 1 [file viruses-17-01264-s001.zip › viruses-3774103-supplementary.pdf]

Table S1. Primers, crRNA, and probe in this study

Name	Sequence (5' -3')
crRNA1	UAAUUUCUACUAAGUGUAGAUUCUGGUGAACUGGCGGCUCC
crRNA2	UAAUUUCUACUAAGUGUAGAUCCCACAGCAACAGGUGCACA
Unrestricted crRNA	UAAUUUCUACUAAGUGUAGAUUCUGGUGAACUGGCGGCUCC
RPA-F1	CCGACAACGCCGAGACTGGTGTTATTGAGGC
RPA-R1	TAACCATCGTCCTGCTGTGCACCTGTTGCT
RPA-F2	CCGACAACGCCGAGACTGGTGTTATTGAGGC
RPA-R2	TCAGACGGGTTGACGTACAGGCCGAAACGG
RPA-F3	CGAGACTGGTGTTATTGAGGCAGGTAACACTG
RPA-R3	TCAGACGGGTTGACGTACAGGCCGAAACGG
ssDNA-FQ probe	FAM-TTATT-BHQ1
KT-DNA-F	GCTCGAGTTATGAATGCGCAAGTTCAGC
KT-DNA-R	TAGTCGACATGGACACTGAAACGTCTCCAC
KT-crRNA	UAAUUUCUACUAAGUGUAGAUGGUAUGCUCGACUUGCAGUC
PRRSV-F	GGCTTGGTTGTTACTGAGACAG
PRRSV-R	GAGGGTACAAGGGGTAACAGTT
CSFV-F	ATGTTTTCTCTTCAAGACATCTGT
CSFV-R	TTACTCACATTTCAATTGAAGTGCT
PEDV-F	GTTTGAAACCAGTAACTGTCGGCT
PEDV-R	AACACCAGTGCCAGCACCAGC
PDCoV-F	CGTGTTACTTGGGTTAAGGGTTCG
PDCoV-R	CAGGAGAACCCTCCTTGACTGTG
SVA-F	AGAATTTGGAAGCCATGCTCT
SVA-R	GAGCCAACATAGARACAGATTGC
SVA qPCR-probe	FAM-TTCAAACCAGGAACACTACTCGAGA-BHQ1

Table S2. Comparison of various CRISPR/Cas12a-SVA detection methods

**Tapes of differences**	**Published two-pot assay[25]**	**Published one-pot assay[24]**	**Two-pot assay (in this study)**	**One-pot assay (in this study)**
RPA step		✓	✓	✓
PCR step	✓			
CRISPR/Cas12a step	✓	✓	✓	✓
Canonical PAM		✓	✓	
Suboptimal PAM				✓
Reaction system	Two	Two	Two	One
Reaction time	> 60 min	40 min	30 min	30 min
Sensitivity (SVA genome)	One copy	Ten copies	Two copies	Two copies
Specificity (No reaction with viruses)	FMDV, PRV, JEV, PPV, CSFV, PCV2, PDCoV, PEDV, ASFV	CSFV, FMDV, PRRSV, PRV, RV, PCV2	CSFV, PEDV, PRRSV, PDCoV	CSFV, PEDV, PRRSV, PDCoV
One-pot method		✓		✓
Two-pot method	✓		✓	
Centrifugation		✓		
Relative reaction cost	High	High	High	Low
